# Academic anaesthesiology: a global perspective on training, support, and future development of early career researchers

**DOI:** 10.1016/j.bja.2023.07.030

**Published:** 2023-09-07

**Authors:** Ottokar Stundner, Meredith C.B. Adams, Jakub Fronczek, Vikas Kaura, Li Li, Megan L. Allen, Emily A. Vail

**Affiliations:** 1 Department of Anesthesiology and Intensive Care, Innsbruck Medical University, Innsbruck, Austria; 2 Departments of Anesthesiology, Biomedical Informatics, Pharmacology & Physiology, and Public Health Sciences, Wake Forest School of Medicine, Winston-Salem, NC, USA; 3 Centre for Intensive Care and Perioperative Medicine, Jagiellonian University Medical College, Kraków, Poland; 4 Leeds Institute of Medical Research at St James's, Faculty of Medicine and Health, University of Leeds, Leeds, UK; 5 Department of Anesthesiology & Pain Medicine, University of Washington, Seattle Children's Hospital, Seattle, WA, USA; 6 Department of Anaesthesia and Pain Management, The Royal Melbourne Hospital and Department of Critical Care, The University of Melbourne, Melbourne, Australia; 7 Department of Anesthesiology & Critical Care, Perelman School of Medicine, University of Pennsylvania, Philadelphia, PA, USA

**Keywords:** academic anaesthesiology, career development, collaboration, funding, mentorship, training

## Abstract

As anaesthesiologists face increasing clinical demands and a limited and competitive funding environment for academic work, the sustainability of academic anaesthesiologists has never been more tenuous. Yet, the speciality needs academic anaesthesiologists in many roles, extending beyond routine clinical duties. Anaesthesiologist educators, researchers, and administrators are required not only to train future generations but also to lead innovation and expansion of anaesthesiology and related specialities, all to improve patient care. This group of early career researchers with geographically distinct training and practice backgrounds aim to highlight the diversity in clinical and academic training and career development pathways for anaesthesiologists globally. Although multiple routes to success exist, one common thread is the need for consistent support of strong mentors and sponsors. Moreover, to address inequitable opportunities, we emphasise the need for diversity and inclusivity through global collaboration and exchange that aims to improve access to research training and participation. We are optimistic that by focusing on these fundamental principles, we can help build a more resilient and sustainable future for academic anaesthesiologists around the world.

**Editor's key points**

• The challenges facing early career academic anaesthesiologists are similar in most well-resourced countries.
• These include long training pathways, variable salary support to provide protected time for academic work, and poor long-term research funding opportunities.
• A focus on mentoring, sponsorship, and diversity is critical to sustaining academic anaesthesiology in the face of overwhelming clinical demands, and to ensure the necessary human capital for innovation and discovery in advancing the specialty to improve patient care.

Anaesthesiology and its related specialities (perioperative, critical care, and pain medicine) have a long history of bold investigations, breakthrough discoveries, and interdisciplinary leadership.^1^, ^2^, ^3^, ^4^ Over the last century, academic anaesthesiologists have laid the educational and organisational foundations to establish anaesthesiology as an independent speciality attracting talented individuals. Although recruitment and development of prospective investigators, educators, and administrators are integral to the speciality's future growth and sustainability, numerous challenges and barriers remain. As a global community, we are responsible for providing a fertile ground for future leaders in anaesthesiology. All authors of this paper are early-career anaesthesiologist researchers, primarily *British Journal of Anaesthesia* Editorial Fellows, and thus well situated to reflect on the current state of academic anaesthesiology and provide perspectives on optimal investigator training and support worldwide.

Careers in academic anaesthesiology are heterogeneous. Although research, teaching, and administration constitute the core of academic anaesthesiology, the proportion of time dedicated to these activities varies across countries, regions, and institutions. Broadly speaking, academic anaesthesiologists are clinicians whose expertise and efforts contribute to the development and expansion of the speciality beyond the provision of routine medical care. Depending on the setting and individual career goals, the focus of an academic anaesthesiologist may be a combination of investigator- or industry-initiated scientific research, supervision of clinical and research training, development of quality improvement programmes, institutional leadership, and policymaking. Achieving eventual success in academia requires the willingness to persevere through setbacks and rejection, which is best fostered through lifelong mentorship and sponsorship. Despite recent initiatives from professional societies and various stakeholders to recognise and develop structured support for academic career development, the road to academic success can appear to be a journey into the unknown, with large differences prevailing between geographic regions (see Table 1). Here, we compare typical academic career paths across selected geographic regions and discuss barriers and opportunities faced by early-career academic anaesthesiologists.

**Table 1:** Formal research training opportunities across the United States and Europe. ACGME, Accreditation Council for Graduate Medical Education; ANZCA, Australian and New Zealand College of Anaesthetists; Aust&NZ, Australia and New Zealand; EACTAIC, European Association of Cardiothoracic and Vascular Anaesthesia and Intensive Care; FAER, Foundation for Anesthesia Education and Research; IARS, International Anesthesia Research Society; MSARF, Medical Student Anesthesia Research Fellowship; NIH, National Institutes of Health: MRC, Medical Research Council; WT, Wellcome Trust; NIHR, National Institute for Health and Care Research.

Type	Region(s) available	Comments
Medical student research programmes	Aust&NZ, EU, UK, USA	Aust&NZ: BMedSci available through some medical schools as a research extension year. Masters and PhD programmes run separately to medical school training. EU: research project as part of MD programme. UK: MB/PhD, MRes, through applying for a PhD at university offering the MB/PhD programme or applying for an MRes course during medical studies. USA: ACGME resident research; FAER MSARF, FAER year out.
Clinical research PhD	Aust&NZ, EU, UK, USA	Aust&NZ: Commonwealth-funded positions available for fee payment. Self-funded or competitive university scholarships for living expenses available, less than medical salary. EU: Institutional Clinical Research PhD programmes offered in many universities as part of the Bologna process. Privately or publicly funded and often combined with clinical work. UK: See ‘Research Training Fellowships’. USA: Self-funded or competitive national and university scholarships for living expenses available.
Clinical research Masters degrees	Aust&NZ, EU, UK, US	Aust&NZ: Part-time/full-time Masters course aimed at clinical research. Privately funded. EU: Part-time/full-time Masters course aimed at clinical research. UK: part-time/full-time Masters course aimed at clinical research (can be included in undergraduate programme, postgraduate would be self-funded/external grant from a charity/part or full institution contribution). USA: Part-time/full-time Masters course.
Basic science PhD	Aust&NZ, EU, UK, USA	Aust&NZ: Commonwealth-funded positions available for fee payment. Self-funded or competitive university scholarships for living expenses available, less than medical salary. EU: Institutional Basic Science PhD programmes offered as part of the Bologna process. Privately or publicly funded. UK: Externally funded by charities/research organisations at nonclinical rate (if undergraduate) or clinical rate (postgraduate, see ‘Research Training Fellowships’) or self-funding/university funded. USA: Self-funded or competitive national and university scholarships for living expenses available.
Research training fellowship	Aust&NZ, EU, UK, USA	Aust&NZ: Available in some academic centres with proportion of time in clinical anaesthesiology generally. EU: Marie Skłodowska-Curie Research Fellowship Program; European Research Council grants for frontiers research; Association-Organised Fellowships (EACTAIC) [usually combined clinical-research]. UK: Clinical Research Training Fellowships MRC/WT/NIHR cover postgraduate doctoral funding in laboratory or clinical research. USA: NIH T32 grant
Research training grants	Aust&NZ, EU, UK, USA	Aust&NZ: Novice investigator grants available from ANZCA. EU: Marie Skłodowska-Curie Actions; European Research Council Training/Starting Grants; Association Grants (ESAIC, National Associations). UK: See ‘Research Training Fellowships’. USA: NIH K series grants, Foundation grants: FAER and IARS resident and junior faculty mentored research grants.

## Academic anaesthesiology training and research funding around the world

### United Kingdom

#### Training

**Fig 1:**
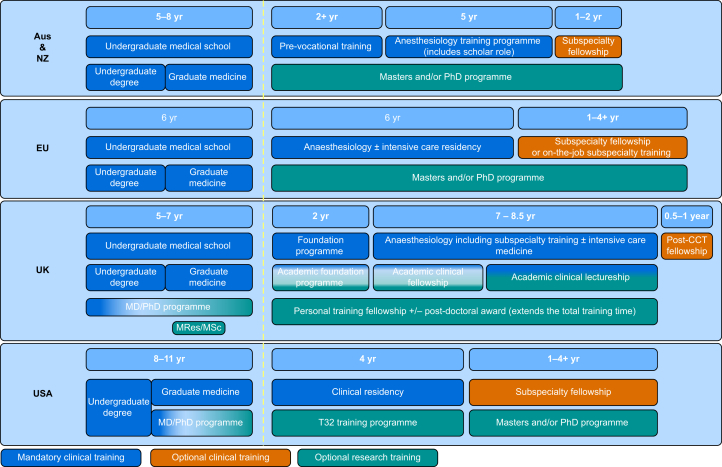
Academic pathways in Australia/New Zealand, the EU, the UK, and the USA. CCT, certificate of completion of training.

The UK medical education system involves 5 yr of undergraduate medical education (or 4 yr for graduates), followed by 2 yr of foundation training to achieve General Medical Council registration (Fig. 1). Anaesthesiology training commences in postgraduate year three and is of 7 yr duration (or 8.5 yr for those seeking dual accreditation with intensive care medicine).^5^ The minimum duration of training is therefore 14 yr from secondary education. Sub-specialisation training occurs during or after the final 2 yr of required clinical training.

Formal research training is not required for full certification in anaesthesiology; during the undergraduate years, interested individuals can complete an intercalated Master's degree programme or formal MB/PhD programme (which award medical and research doctorates after 7 yr of training). Up to 6 months near the end of postgraduate training can be used to gain experience in research, quality improvement, or medical education. Informal clinical research experience can be gained throughout training by participating in collaboratives such as the Sprint National Anaesthesia projects^6^^,^^7^ or the Research and Audit Federation of Trainees.^8^^,^^9^

#### Funding

Individuals interested in postgraduate research training have formally funded research opportunities, but these vary within the UK; a majority are in England through the Integrated Clinical Academic Pathway. This programme operates in parallel with the standard clinical training programmes and requires a separate competitive application for each training stage. The National Institute for Health and Care Research (NIHR) academic foundation programme funds 3–6 months of academic time over the 2-yr foundation training.^10^ Next is a 3-yr NIHR Academic Clinical Fellowship which, in addition to anaesthesiology training, funds 25% of protected academic time to develop competitive applications for externally funded research training fellowships from organisations such as the Medical Research Council, Wellcome Trust, NIHR, or charities. These prestigious fellowships fund the individual's salary, university fees, and research costs allowing them to undertake full-time doctoral research training over 3–4 yr, while temporarily suspending their clinical training. Investigators not supported by research training fellowships can find support for degree completion through project or programme grants, private hospitals, or host institutional funds.

The final stage of the integrated clinical and academic pathway is a 4-yr NIHR Academic Clinical Lectureship post, which funds both clinical training and academic time of up to 50%. During the lectureship, trainees complete clinical training and develop proposals for postdoctoral research training fellowships of 3–7 yr duration, after which they would become senior independent extramurally funded researchers with a substantive university appointment. In other cases, clinicians with higher degrees can be supported by dual hospital and university contracts.

### United States of America

#### Training

In the USA, training in clinical anaesthesiology requires a minimum of 12 yr after secondary school graduation. This consists of 4 yr of undergraduate education and 4 yr of medical school to obtain an MD. Many medical schools offer combined MD/PhD programmes with a duration of 6–8 yr.^11^ Anaesthesiology residency encompasses 12 months of fundamental clinical training in medicine or surgery and 36 months of training in clinical anaesthesiology (Fig. 1). Although the Accreditation Council for Graduate Medical Education permits 6 months of research during the third year of anaesthesiology training, only 28 of 105 accredited residency training programs currently offer specialised tracks that integrate research training and protected time into clinical residency.^12^, ^13^, ^14^ The NIH National Center for Advancing Translational Sciences Center provides additional opportunities for institutions to support trainee and faculty research training and skills development by funding programmes that support collaborative science, remove research barriers, and facilitate research skills training (whether certificate or higher degree programmes).^15^

#### Funding

Several bridge funding options after residency provide protected academic time and additional research training needed for development of independent investigators. These include National Institutes of Health (NIH) T32 programmes at select institutions, NIH K series grants or individual mentored research training grants, and grants from private foundations such as the Foundation for Anesthesia Education and Research (FAER) and the International Anesthesia Research Society (IARS). Intended for physicians without prior formal research training, NIH T32 programmes provide 2–3 yr of substantial protected research time (80%) and are currently available in 16 anaesthesiology departments in the USA.^16^ After research training and early funding focused on career development, the long-term goal for most academic anaesthesiologists serving as principal investigators in investigator-initiated research projects is to secure sustained funding for competitive grants from the NIH and other federal agencies to support individual research projects and cooperative programs. Others may support individual research projects through charitable foundation grants or industry sponsorship.

Academic medical centres in the USA recognise fundamental differences in daily responsibilities and academic productivity between clinical faculty and those with heavier research designations. As a result, many utilise a different system of promotion and tenure for clinical faculty, broadly organised into separate tracks for primary clinicians and administrators, clinician educators, and researchers including clinician-scientists engaged in collaborative or investigator-initiated research. Such systems encourage faculty to focus on specific professional competencies (e.g. healthcare management or curriculum development) and recognise that an individual's contributions to academic medicine extend beyond the traditional paradigm of extramurally funded, investigator-initiated basic science research.

### Continental Europe

#### Training

Medical schools in continental Europe traditionally offered a 6-yr undergraduate medical degree^17^ (Fig. 1). The Bologna process was an agreement introduced in 1999 to reconcile higher education pathways across the European Union, therefore facilitating mobility and degree alignment between countries.^18^ Consequently, multiple countries introduced combined undergraduate and graduate degree pathways with some including research components. An increasing number of medical schools also offer combined MD/PhD degree programmes. However, the planning and implementation of the Bologna process remain controversial; some have argued that it has done little to unify European medical education to date.^19^ Considerable differences in residency training models also exist between European countries. Most anaesthesiology residencies consist of a 5- or 6-yr program immediately after medical school, with a varying mixture of formalised and informal experiential training. In some countries, research time spent towards a full doctoral degree or PhD is recognised. The overwhelming majority of postgraduate (post-residency) positions available are clinical, with some employers offering a varying, usually small, fraction of non-clinical research or administrative time. Research-only positions are limited to universities and large university-affiliated hospitals with established research infrastructure. Clinical sub-specialisation occurs between the end of residency and during the first years as a junior specialist. In most countries, no formal fellowship system exists, neither for clinical nor research fellowships, and sub-specialisation is achieved through supervised exposure to clinical cases. For clinician-scientists, access to research groups, resources, time, and funding is often organised in a similarly informal way, through personal communication within departments, specialised groups, meetings, or peer networks. Lack of established routes to, or barriers to entry into, these groups can be a major obstacle for medical students and junior graduates to gain access to research despite their interest. The Section and Board of Anaesthesiology of the European Union of Medical Specialists, an organisation loosely affiliated with the European Commission, publishes a list of European Training Requirements in anaesthesiology and intensive care along with a list of ‘generic competencies and roles’ for anaesthesiologists. These include the role of an ‘Academic scholar’, defined as an individual who knows, critically appraises, and contributes to current literature, teaches both clinical, research, and academic presentation skills, and serves to promote the speciality itself.^20^ Complementary to this theoretical framework, the European Society of Anaesthesiology and Intensive Care (ESAIC) provides practical resources to achieve some of these goals across Europe. Among these resources are trainee exchange programmes, unified certification (the European Diploma in Anaesthesia and Intensive Care [EDAIC]), courses, a clinical trial network, research mentorship programmes, and research grants.^21^

#### Funding

Most medical research in Europe is conducted in state or municipal universities, which are largely financed through political channels that are independent of individual projects.^22^ Thus, it is uncommon for junior doctors to have to provide their own funds when entering junior researcher positions; however, they may have to commit to securing third-party funding within a certain amount of time as a requirement for tenure and/or long-term employment. Project-based funding is available at the level of individual institutions, municipalities, counties, and institutions, and at the level of the EU. All of these have different eligibility criteria, rules, and application processes. To name only the most important grants available to all European researchers, the EU provides medical funding up to 2.5 million euros through the European Research Council's (ERC) starting, consolidator, advanced, and proof-of-concept grants^23^; the Marie Skłodowska-Curie Actions (MSCA), funding doctoral and postdoctoral trainees^24^; a broad funding project entitled Horizon Europe^25^; and through numerous smaller-scale agencies, such as digitalisation or environmental responsibility.^26^ Common to all these programmes, as well as to many national programmes, is that they are available to all disciplines, signifying that applicants are broadly competing with scientists from all fields of research. Several anaesthesiology societies attempt to fill gaps in the funding landscape by offering grants and prizes, some aimed at funding junior researchers. These include but are not limited to: *BJA* Foundation-ESAIC Grants for early career development^27^; ESAIC project grants (available individually for anaesthesiology or intensive care); European Society of Regional Anaesthesia and Pain Therapy grants^28^; and European Association of Cardiothoracic and Vascular Anaesthesia and Intensive Care (EACTAIC) grants.^29^ Most national anaesthesiology and intensive care societies provide similar funding opportunities within individual European countries.

### Australia and Aotearoa New Zealand

#### Training

The last two decades have brought a shift in medical school training in Australia and New Zealand from an exclusively 6-yr undergraduate course to a combined undergraduate and postgraduate pathway with 4- or 5-yr options (Fig. 1). This has resulted in some medical school graduates holding a science qualification or even a research higher degree before undertaking their specialist anaesthesiology training.

Completion of training in anaesthesiology takes a minimum of 7 yr after medical school: 2 yr of general hospital experience followed by 5 yr of anaesthesiology training. The Australian and New Zealand College of Anaesthetists (ANZCA) training program includes seven core domains, and one of these termed the ‘scholar role’ requires skill development in reviewing and critically appraising the literature, clinical audit, and teaching others.^30^

Further discretionary research training (e.g. Master's or Doctoral degree programmes) can be undertaken before, during, or after anaesthesiology training. There is currently no dedicated academic anaesthesiology training pathway that integrates a research qualification with anaesthesiology training. Although additional research training is not mandated, it is increasingly difficult to secure competitive grant funding without a higher degree.

The ANZCA Clinical Trials Network (CTN) supports investigator-initiated and -led multicentre trials in the domains of anaesthesiology, pain, and perioperative medicine.^31^ The ANZCA CTN has completed many large, impactful trials addressing pragmatic research questions.^32^^,^^33^ They foster development of emerging anaesthesiology researchers including a skills development programme during the annual ANZCA meeting, mentorship if required, and inclusion of emerging investigators on major competitive grant funding applications. In addition, emerging anaesthesiology investigators can gain research leadership experience by acting as site investigators for CTN-endorsed multicentre trials at their hospital to gain experience within a supported structure and lead a sub-study arising from a large trial.

#### Funding

The remuneration of anaesthetists in Australia and New Zealand is commonly through the performance of clinical anaesthesiology. Most university academic appointments are honorary or are minimally paid compared with the income earned through clinical anaesthesiology. Therefore, academic anaesthesiologists including those with a university appointment typically maintain a substantial clinical caseload. A survey of anaesthesiologists across Australia and New Zealand found that the characteristics associated with anaesthesiologists intending to undertake research in the future included having research qualifications or planning to obtain research training, previous research involvement, and obtaining their fellowship in the last 5 yr.^34^

The ANZCA has a foundation that annually provides seed funding to ANZCA fellows and trainees in Australia and New Zealand. These grants are typically aimed at pilot and feasibility work to underpin a larger competitive grant application. The ANZCA foundation has specific emerging investigator grants to support early career researchers. The National Health and Medical Research Council (NHMRC) is Australia's primary research funding body, and the Health Research Council (HRC) serves this role in New Zealand. From 2013 to 2022, anaesthesiology research received only $38.7 million (Australian) in NHMRC funding from a total funding allocation of $2.9 billion (Australian) for clinical medicine and scientific research and $3.4 billion (Australian) for basic science research. Anaesthesiology research funding in New Zealand through the HRC has been similarly low with a total spend of less than $1 million (New Zealand) over the last 5 yr representing 0.15% of the total research budget allocation. In 2021, anaesthetists comprised 6.8% of the medical specialist workforce in Australia^35^ and 8.3% in New Zealand.^36^ Therefore, anaesthesiology research has been relatively poorly funded compared with other medical specialities. In Australia, a new funding stream, the Medical Research Future Fund (MRFF) was established in 2015.^37^ This government investment fund receives external consultative advice to determine funding priorities. Several large grants have been made to anaesthesiology research since its establishment, which is promising for future substantial funding for anaesthesiology research.

## Equivalence in training and mobility

Exchange programs can be an effective way for academic anaesthesiologists to gain knowledge and experience, broaden their perspectives, expand their networks, overcome local limitations in training and experience, and bring new expertise back to their institution and country. Researchers, particularly those from Europe, frequently spend a year or more participating in research projects abroad. However, the organisational and regulatory differences between countries are common barriers. Moving abroad for research is contingent on the availability of a sponsor, visas and work permits, funding, and personal circumstances. Additionally, those planning to perform clinical work abroad (either to gain experience or to support their salary) can require additional examinations, certifications, or further clinical training. As a result, opportunities to participate are less feasible for potential trainees with fewer personal resources, working in low-income countries, and/or with personal caregiving responsibilities. Given the enormous professional advantages offered by international exchange programs, systems are needed to address barriers to access and make opportunities for exchange more equitable across regions and countries.

## Current challenges, solutions and unique opportunities

Several major global challenges remain in academic anaesthesiology. These include barriers to accessing research opportunities, lack of incentives, lack of funding, and lack of diversity. We have identified several broadly applicable opportunities to overcome some of these barriers, including *mentorship*, *collaboration*, and *digitisation*.

### Barriers to an academic anaesthesiology career pathway

The report on a National Strategy for Academic Anaesthesia in 2005 highlighted the severe crisis in academic anaesthesiology that was occurring in the UK at the time,^38^ and similar occurrences were reported from other parts of the world.^39^ This decline has continued despite strong efforts to reverse it, with further losses in academic departments.^40^ This has led to reduced opportunities for formal training in anaesthesiology research, resulting in fewer individuals pursuing academic anaesthesiology. Furthermore, underfilled consultant jobs^41^ reduce the incentive for anaesthesiologists to invest in formal research training to enhance their career prospects; this contrasts with certain surgical specialities, particularly those in tertiary hospitals.^42^^,^^43^ These factors have led to clinical trainees seeking to gain the research exposure necessary to meet the requirements of their training programme working within a more collaborative research model of recruiting to large studies, or those run by trainee research networks.^9^ Although these provide an element of research exposure, they generally do not allow individuals to get any formal or in-depth training in research.

### Lack of funded research time

In a 2016 survey of 2000 physicians in the UK, difficulties in applying for and receiving protected time and funding was cited as the most important barrier to conducting research.^44^ Anaesthesiologists already involved in research or intending to undertake research in the future spend more of their time in public hospitals (government funded) rather than private (insurance reimbursed) practice. In some geographical regions, particularly those with a larger proportion of project-independent university funding, public hospital anaesthesiology appointments include a component of paid non-clinical time for academic work. Non-clinical academic time is variable but is commonly half to one day per week if working full-time in Europe, Australia, or Aotearoa New Zealand; such time is uncommon in the USA and UK without specific research-funded salary support. Non-clinical time is usually unavailable in private anaesthesiology practice, where salaries are more directly tied to clinical productivity. For healthcare systems, the generation of scientific results or achievement of individual or collective academic goals cannot be translated into short-cycle economic gains. Moreover, clinical employers are burdened by recurrent economic crises and concerns about the impending failure of healthcare systems.^45^ In summary, there is universal tension between time spent generating income practising clinical anaesthesiology and poorly funded or unfunded academic time.

Although self-sustainability in academia is the goal, funding sources remain limited, competitive, and heterogeneous. Additionally, academic anaesthesiologists typically require several additional years of specific research training; lower salaries during prolonged training periods significantly disincentivise this career path. The disincentive can be amplified for individuals with debt, and those with financially dependent family members or caregiving responsibilities at home.^46^, ^47^, ^48^

Universities, irrespective of whether they are privately or publicly funded, rely on prestige to retain and attract income. This prestige can take many forms, including endowment, scientific impact, citation metrics, and, importantly, funding attracted, in a feed-forward cycle. Research institutions seeking high-impact science require large upfront and ongoing investments in research infrastructure. Much of the contemporary funding landscape is outsourced, competitive, results-oriented and, by extension, merit-oriented. To attain results and merit, junior researchers require considerable institutional investment to reach academic independence and self-sufficiency. From an economic perspective, funding of junior investigators is uncertain and carries risk, which is a direct disincentive to investing in resources required to support early careers.^49^ As entry into research becomes increasingly competitive, individuals privileged with early opportunities for success are differentially supported.

Despite the role of anaesthesiologists as innovators and drivers of technological advancement,^50^ opportunities for the development of junior researchers remain scarce. Perceptions about unfavourable compensation, professional responsibilities outside of clinical duties, and concerns about job security (whether promotion and tenure or sustained research grant support) steer most trainees into more remunerable positions such as private practice, and more stable roles as full-time clinicians, medical educators, or administrators in academic medical centres.^51^

### Lack of diversity

Diverse teams in healthcare are associated with clinical excellence,^52^, ^53^, ^54^ but diversity has not been achieved in anaesthesiology.^55^^,^^56^ Although the problem is recognised and mitigation initiatives exist, systemic barriers to diversity remain.^57^ Prolonged periods of training, insufficient early career research funding, and differential salaries in academic compared with private practice anaesthesiology place a burden on those from disadvantaged socioeconomic, gender, and cultural groups, precisely those currently underrepresented in academic anaesthesiology, yet necessary to build performance and innovation.

Despite increasing parity among students and recent graduates, faculty researchers are less likely to be members of underrepresented groups,^58^ who disproportionately face systematic disadvantages, overt discrimination, lack of mentoring or representative role models,^59^ insufficient compensation to overcome generational wealth gaps, and lack of social support structures (such as organised child care and family care^60^). Although formal physician-scientist training programmes (e.g. combined MD/PhD training) offer free tuition and stipends for accepted students, the positions require early career differentiation and are highly competitive (favouring applicants from major research universities with early research experience and supportive mentors) than programmes offering individual clinical medical degrees.

Most advocacy groups and societies are committed to achieving gender equity and actively report on this issue (e.g. ANZCA,^61^ the Society for Pediatric Anesthesia,^62^ and cardiothoracic anaesthesiology societies^63^). In Australia and New Zealand, only 33% of specialist anaesthetists were female, which was even lower for those close to retirement age despite 43% of trainees being female. Reassuringly, a female lead was listed for 44% of research grants, an increase of 9% since the last report. However, progression to academic leadership is challenging as only 12.5% of full professors in Australia and New Zealand are women, with similar situations reported from other parts of the world.^64^ Unfortunately, there is a lack of such reporting based on cultural, socioeconomic, and carer roles regardless of gender.

Pipeline remedies are slow and incomplete. Some targeted mitigation strategies for lack of diversity include investment in research infrastructure at minority-serving institutions,^65^ or establishment of specialised agencies such as the US NIH Office for Scientific Workforce Diversity.^66^

## Conclusions and recommendations

Despite the challenges and barriers to pursuing an academic anaesthesiology career, we remain hopeful that the community will develop and support current and future generations of clinician-scientists. Our specific 12-point recommendations for the future of academic anaesthesiology training are summarised in Table 2.

**Table 2:** Recommendations for the future of academic anaesthesiology training. ANZCA, Australian and New Zealand College of Anaesthetists; ESAIC, European Society of Anaesthesiology and Intensive Care; FAER, Foundation for Anesthesia Education and Research; IARS, International Anesthesia Research Society.

No.	Recommendation	Description of recommendation
1	***Expand the scope of academic anaesthesiology***	Include roles and competencies vital to innovation and contemporary anaesthesiology practice
2	***Emphasise the importance of research***	Foster an explicitly stated institutional research culture,^67^ invest in resources to support high-quality research; reward individuals who are participating^68^
3	***Develop and advertise innovative educational programmes***	Research training shown to be highly effective when delivered in integrated programmes, such as dedicated research fellowships^69^^,^^70^
4	***Increase interdepartmental/interdisciplinary collaboration and research***	Involve external specialists (e.g. statisticians or public health researchers) early on in research projects, foster collaborations across disciplines that intersect with anaesthesiology (e.g. surgery, intensive care, internal medicine/paediatrics, and pain management) to achieve more comprehensive treatment strategies^71^^,^^72^
5	***Focus on patient-centred care and patient-important outcomes***	Healthcare continues to shift towards a more patient-centred approach; prioritise patient-centred care in research, education, and clinical practice^73^; in basic and other non-clinical research, emphasise translational aspects
6	***Embrace diversity, equity, and inclusion***	Actively promote diversity, equity, and inclusion, both in clinical practice, research, administration, and education: recruit and retain a diverse workforce; address disparities through identification and research; emphasise inclusivity in research; embed diversity requirements for competitive research funding grants^74^
7	***Improve access to funding***	No universal solutions to lack of funding issues; complexity requires individualised approaches: advocacy efforts with policymakers and stakeholders; highlight the significance of anaesthesiology research and its potential impact on patient care; embrace collaborative research efforts to unlock larger funding opportunities
8	***Examine other systems and learn from each other***	Learn from other systems (e.g. the UK and USA offer more standardised training pathways and training-oriented funding than Continental Europe); EU should continue to expand its role as organiser and sponsor of integrated research programmes; in Europe, entry into research can often be accomplished without external resources through project-independent university funding, but knowledge and skills to attract competitive funding at a later stage are not part of many training curricula; in terms of collaboration, the ANZCA has proven that trial networks can be implemented even in comparatively low-population countries and across diverse geographical locations
9	***Explore future directions and new skills for academic anaesthesiologists***	Work in teams with diverse skill sets (data science, public health-based and population level approaches, large multidisciplinary research groups, implementation and healthcare delivery science, quality improvement science, and global health^75^, ^76^, ^77^) could alleviate the training burden for individuals while harnessing the befits of these varied methodologies
10	***Leverage technology***	Use tools that improve and simplify collaboration (e.g. shared document editing, project management tools, video conferencing); leverage improved accessibility, dissemination, and much larger audience for research results (e.g. preprints, transparent peer review)
11	***Improve access to and actively advertise research collaborations***	Join multicentre studies as a centre; serve as local principal investigators for multicentre studies or contribute data to snapshot audit studies^78^, ^79^, ^80^; utilise international education and research collaboration groups (including FAER, IARS, ANZCA, ESAIC, specialist societies, and anaesthesiology and intensive care societies); be a local champion to advertise and implement such projects
12	***Improve research literacy***	Implement mandatory or elective research elements in training programmes.

Although individual challenges and barriers to a fulfilling and sustainable academic anaesthesiology career are common and expected, recognising systemic issues limiting career entry, growth, and retention is needed to develop and translate innovative solutions from healthcare systems around the world. Highly successful research careers are challenging endeavours in themselves; convincing institutions and healthcare systems to support individuals working on these pathways should not be the primary barrier. Although current systems of funding are increasingly challenging, academic departments should try to support the skills and time for trainees and junior faculty who are committed and wish to embark on this path. Although funding issues will inevitably persist, we recommend establishing a strong network of mentors, recognising and addressing sources of inequity, and pooling resources and expertise across institutions and countries to empower the next generation of academic anaesthesiologists ready to transform the speciality anaesthesiology.

## Authors’ contributions

Written sections of this manuscript, reviewed and edited the manuscript draft, reviewed and revised the final manuscript before submission: all authors.

## Declarations of interest

OS, MCBA, EAV, JF, VK, and LL are former editorial fellows, and EAV and JF are current members of the associate editorial board of the *British Journal of Anaesthesia*. The authors declare no other conflicts of interest.

## Funding

US National Institutes of Health (K08 EB022631 to MCBA); US Agency for Healthcare Research and Quality (K12 HS026372-04 to EAV). The content is solely the responsibility of the authors and does not necessarily represent the official views of the Agency for Healthcare Research and Quality.
